# Transitioning Out of the Coronavirus Lockdown: A Framework for Evaluating Zone-Based Social Distancing

**DOI:** 10.3389/fpubh.2020.00266

**Published:** 2020-06-08

**Authors:** Eric Friedman, John Friedman, Simon Johnson, Adam Landsberg

**Affiliations:** ^1^International Computer Science Institute and Department of IEOR, University of California, Berkeley, Berkeley, CA, United States; ^2^COVID-19 Policy Alliance, Cambridge, MA, United States; ^3^Department of Statistics, UC Berkeley, Berkeley, CA, United States; ^4^Sloan School of Management, MIT, Cambridge, MA, United States; ^5^W.M. Keck Science Department, Claremont McKenna College, Claremont, CA, United States; ^6^W.M. Keck Science Department, Pitzer College, Claremont, CA, United States; ^7^W.M. Keck Science Department, Scripps College, Claremont, CA, United States

**Keywords:** covid-19, zones, reproductive number, networks, social distancing measures, SIR (Susceptible Infected-Recovered) model

## Abstract

In the face of elevated pandemic risk, canonical epidemiological models imply the need for extreme social distancing over a prolonged period. Alternatively, people could be organized into zones, with more interactions inside their zone than across zones. Zones can deliver significantly lower infection rates, with less social distancing, particularly if combined with simple quarantine rules and contact tracing. This paper provides a framework for understanding and evaluating the implications of zones, quarantines, and other complementary policies.

## Introduction

As a result of its implications for health and mortality, the Coronavirus disease (COVID-19) pandemic has triggered massive disruptions to both economies and social structures (1). In addition to more than 2 million infections and over 120,000 deaths worldwide by mid-April 2020 (2), the resulting widespread lockdowns have depressed economic activity and sharply reduced the income of many people (3). In the United States, GDP will decline by at least 7 percent in the second quarter of 2020, and unemployment is expected to exceed 10 percent (4). The speed of this decline in measured economic activity is also dramatic: in 1 week, the number of new unemployment claims was 10 times larger than in any single week of the 2007-08 recession (5). By the second week of April 2020, unemployment in the United States was already around 13%, the highest rate since the Great Depression (5).

The effect on human relationships is also unprecedented, with people effectively dissuaded from seeing friends in person and forbidden from visiting loved ones in senior care facilities (6). The impact of lockdowns on individual health is also likely to prove significant (7).

While the precise future course of infection is debated, leaders in many countries have begun to think about how best to transition out of the complete lockdown phase (8). However, with good reason, the World Health Organization warns that abruptly ending comprehensive “stay at home” orders could result in new outbreaks (9), and the Centers for Disease Control and Prevention (CDC) is concerned there may be at least one more wave of infection in late fall or early winter (10). Consequently, a number of prominent analysts (11–13) have emphasized that social distancing should be relaxed in various gradual ways. On April 16, 2020, guidance issued by the White House seemed to support that general notion—while placing the onus on governors to decide the details (14).

At the same time, some companies are dividing their workforces into non-intersecting groups (15), and governments are reopening some spaces for physical activities, such as walking on the beach (16). How should we think about the properties of this emergent structure of economic and social zones, relative to the spread of disease? Comprehensive stay at home policies are being relaxed, but what exactly should replace them?

We present a simple epidemiological model of “zonal social distancing” that offers a framework for assessing the efficacy of zone-based policies. This is done using an SIR epidemic model on a network with defined zones, from which we can compute an “inter-zonal reproduction number” to quantify its effectiveness and potentially help manage disease progression. The model highlights the potential advantages of organizing people into zones (i.e., a particular structure of groups) such that there are strong interactions within each zone but weak interactions across zones. It also illustrates the value of self-quarantine rules within zones.

Our analysis is meant to be a germane addition to the large body of work on the dynamic spread of infectious diseases (17–19) and particularly the spread of COVID-19 (20, 21) and to provide an additional tool in the design of targeted social distancing policies (22).

## Inter-Zonal Reproduction Number

A long-established idea is that the behavior of any epidemic depends crucially on the basic reproduction number, R_0_, which is the expected number of people that an infected person infects, when the entire population is susceptible. If R_0_ <1 then the epidemic will typically die out after infecting only a small number of nodes, while if R_0_ > 1 then the epidemic is likely to spread widely, infecting a significant fraction of the population.

We extend this idea and compute R_Z_, the inter-zonal reproduction number, which captures the interaction between how zones are structured and—crucially—the speed with which people within a zone can be separated from other zones should an infection enter that zone. As with R_0_, if R_Z_ < 1, then it is likely that only a small number of the zones will become infected and need to be isolated from each other, while if R_Z_ > 1 then it is likely that a larger fraction of the zones could become infected. This follows from the same analysis of the basic reproduction number and is discussed in more detail in the Technical Appendix. Thus, the goal of *zonal social distancing policies* should be to ensure R_Z_ < 1.

The fundamental equation for R_Z_ can be written as:

$$\begin{array}{l} R_{\text{Z}}=R_{0}T_{\text{R}}C_{\text{R}}I_{\text{T}} \end{array}$$

The three parameters of interest are: the “truncation ratio” (T_R_), which captures the ratio of social contacts under a zonal social distancing policy, relative to the unconstrained social structure (i.e., no distancing requirements or social pressures of any kind); the “inter-zonal connectivity ratio” (C_R_), which measures the ratio of interzonal social contacts to total contacts; and the “external infectivity time” (I_T_), which captures the speed and effectiveness with which a zone can be isolated from other zones, when necessary (See the Technical Appendix for an illustrative calculation implying that I_T_ is likely to be order 1 for reasonable parameters).

## Motivating Example

Imagine 50 families, each comprised of two people living together in the same neighborhood. When a pandemic threatens, policy aims to reduce R_0_ below 1, with the goal of slowing down the rate of infection, a process known informally as “flattening the curve.” Without zones, the only way to do this is with a low truncation ratio, so people are told to “shelter in place,” which in this example means that each family stays in its house to the fullest extent possible—everyone is told to eliminate physical contact, if feasible, with anyone outside the home.

With a zonal structure, the situation is different. For example, people could be divided into 10 zones, each containing 5 families. If these families interact primarily within their zones, this would imply a low value of C_R_, so R_Z_ could be less than one without need to resort to shelter in place. This can still work even with imperfect enforcement of the zonal restrictions.

A further major benefit of zones appears if, when an infection enters a zone, everyone in that zone immediately goes into quarantine in their own home. When there is faster self-quarantining for family groups once an infection enters a zone, I_T_ is lower. For example, one could lock down a zone as soon as a patient tests positive for Covid-19 or even when a person first shows symptoms of the virus even before testing in some situations.

In effect, if the agreed goal of policy is to attain R_Z_ < 1, this can be achieved either with shelter in place for everyone (low T_R_), or through a combination of zonal and quarantine policy, which lower C_R_ and I_T_ respectively.

We next discuss the extent to which zones are already emerging, then describe our formal model, and lastly discuss several potential scenarios which illustrate the importance of interaction effects—in a way that can help guide employers, government, and other relevant decision-makers.

## Advantages of Zone-Based Social Distancing

One simple and potentially effective zone-based approach would be to allow people to go to work and interact in person as necessary, but to limit non-work physical interactions to their own household. A version of this has been enacted in parts of Germany (23, 24). Our analysis suggests that allowing social interactions between people whose family members (living in the same house) already work together would not significantly increase risk, as long as this does not also increase connections between zones.

Further compartmentalizing within companies can increase resilience. For example, a company could divide employees into non-overlapping shifts, which are forbidden from meeting in person in any context. The U.S. Centers for Disease Control and Prevention (CDC) recommends staggered shifts, which are at least consistent with this notion (25).

Another specific form of zonal social distancing involves isolating towns or cities from each other, while allowing interactions within them, as in recent Italian travel restrictions (26). One zone-based policy for a city might still allow some people to go to work in nearby cities, but would also cutoff such inter-zonal interactions at the first signs of an infection in the city.

While zones structured around employment are important for economic activity, other zones could be designed to improve social and mental well-being. For example, older citizens who are self-isolating could be allowed to interact with people of their own age in small groups, but not with anyone else. Similarly, families with young children could interact with each other or with dedicated (intra-zone) childcare providers, but not with people outside their zone.

This list is not meant to be exhaustive as many other zonal strategies can and should be considered, based on the objective. One key point is that any movement of non-immune people across zones should be accompanied by comprehensive testing and appropriate quarantine periods. Such decisions are likely to depend on the details of the situation, so the goal of this paper is to provide tools to allow such decisions to be analyzed.

There are also some important caveats and limitations to any zone-based approach. People who are at elevated risk of death should be isolated as much as possible, either in family groups or—once there has been sufficient testing—in small social groups. A zonal structure can reduce the overall rate of infection, which is an important goal of existing policy, but when a single infection is likely to cause death, additional safeguards are surely warranted (27). Wearing masks in public and keeping a safe distance from strangers—standard tools to reduce R_0_–remain very important, even if a zone structure is in effect.

## SIR model on a Zonal Network

We now present an informal overview of our model and the main results. The formal analysis is in the Technical Appendix in Supplementary Material.

We consider a standard stochastic Susceptible-Infected-Removed (SIR) network model (28). That model considers a random network with n nodes and an average of d neighbors for each node. It starts with most nodes in the S (susceptible) state with the remaining nodes in the I (infected) state. Then, in every period, each infected node infects some of its neighbors. Infected nodes are removed (R) after an average of T time. The total probability that such a node infects its neighbor over that time period is q.

We extend the standard model to an SIR model on a random “zonal network.” This network is divided up into m zones, where each zone contains n/m nodes. The average number of neighbors of a given node is d = d_i_ + d_o_, where d_i_ is the number of internal connections (to nodes in its own zone) and d_o_ refers to inter-zonal connections. A key parameter is the “inter-zonal connectivity ratio” C_R_ = d_o_/d, where smaller values of C_R_ correspond to less interaction between zones.

Next, we consider zonal-social-distancing. When it is determined that there are infected nodes in a zone, that zone is isolated—in the sense of having no further interactions with other zones—and everyone in the zone goes into self-quarantine (i.e., disconnects from everyone else as much as possible). The goal of this procedure is to minimize the number of additional zones which will become infected, as well as to reduce the spread of disease within the zone. A second key parameter is therefore the “external infectivity time,” I_T_ = (t_1_+t_2_+…t_k_)/T where t_i_ is the amount of time the i'th infected individual (out of k total) was contagious before the zone was locked down.

Analogous to the basic reproduction number R_0_, we construct an “inter-zonal reproduction number” R_Z_, which is the expected number of other zones that will become infected from an infected zone before it was isolated. As discussed earlier, if R_Z_ < 1, then it is likely that only a small number of the zones will be infected and need to be isolated, while if R_Z_ > 1 then it is likely that a large fraction of the zones will need to be isolated.

Using our model, we show that

$$\begin{array}{l} R_{\text{Z}}=R_{0}T_{\text{R}}C_{\text{R}}I_{\text{T}} \end{array}$$

which we discussed earlier.

## Policy Options and Implications

To demonstrate the value of zone-based social distancing, and the pitfalls that may arise if it is done incorrectly, we offer the following example.

Consider a (non-zonal) social-distancing policy in which people are allowed to socialize within D city blocks of their homes and compare that to a zonal-social-distancing policy where we divide the region into zones of 2Dx2D squares and require people to stay physically in their zone. Assuming full compliance with the zonal policy, we see that C_R_ = 0 and R_Z_ = 0. However, even assuming full compliance with the distance-based policy there would be a significantly larger value for R_Z_, since chains of contacts could extend many miles. With only partial compliance, the zonal policy is still likely to be significantly more effective.

We simulated this simple example, assuming people violate the zone rules 2% of the time, by interacting with someone in another zone (Details are in the Technical Appendix). Using reasonable assumptions for parameters, we found that the distance-based method of separating people fails to contain the virus within D city blocks about 27% of the time, while the zone-based social distancing only fails about 6% of the time and in this case we compute R_Z_ = 0.427 which is significantly less than R_0_ = 5. We would need to enlarge the area of the isolation region by a factor of about 2.5 in the distance-based policy to match the effectiveness of the zonal policy.

## Actionable Recommendations

Intuition based on our model can be applied in various ways. For workplace-based zones, the internal connections are between employees in the same company, while the most important external connections typically arise via people with whom the employees live, especially those who work in other companies (or for any other employer). One insight here is that if a company discovers a single infected employee within one of its zones (e.g., shifts), then the company could immediately tell all employees in that zone to quarantine at home. In addition, everyone living with any worker-thus-quarantined should themselves also go into quarantine, to prevent the disease from spreading to other companies.

While zonal social distancing slows spreading between zones, it also allows for simple quarantine policies within a zone, and these can support standard public health measures. For example, since self-quarantine within a zone is far less onerous from a broader social perspective than a widespread (or country-wide) quarantine, it can be initiated at the first sign of infection within that zone. Importantly, this could also significantly reduce the complexity of contact tracings as the vast majority of everyone's contacts would be in a single zone. In addition, one could potentially combine zonal policies with pooled testing (29, 30), which allows an entire zone to be tested, while lowering the usage of reagents that may be in short supply (31).

One could add a dynamic element to our analysis by assuming random infections spring up within zones, arising from interactions outside our model. One could use these to “flatten the curve” in a controlled dynamic fashion. For example, when hospitals are reaching capacity, a planner could pre-emptively isolate some zones, thereby reducing the number of potential new (random) infections. This would also lower R_Z_, thereby reducing the probability of a large epidemic. Alternatively, as the number of cases ebbs, one could merge zones or gradually increase the number of allowed inter-zonal interactions.

## Conclusions

Many governments have recently begun or are planning to transition out of complete lockdowns. The use of zone-based policies could be used to regulate and “soften” this potentially abrupt transition by both reducing the zonal reproduction number and allow for rapid zonal lockdowns should problems arise. As discussed earlier, many lockdowns in a variety of countries are effectively zone-based; consequently our analysis can clarify and assist in the tuning of such policies. In particular, computing the zonal reproduction number (R_Z_) can be helpful for developing and evaluating such policies in a variety of settings. Indeed, the broad concept of a zonal reproduction number proposed here could serve as a useful metric for managing zonal lockdowns since it is constructed on the basis of very general considerations. Nonetheless, caution is warranted, since there exist many potential varieties of zone-based policies and there are a number of subtle yet critical issues involved in their implementation that will need to be taken into account, including issues of equity, protection of essential workers and economically disadvantaged workers, and protection of individuals with special risk factors. Nonetheless, while zone-based policies will not always be feasible, the analysis and tools developed here can potentially provide critical quantitative insights that can inform and better manage lockdown policies.

## Author Contributions

All authors listed have made a substantial, direct and intellectual contribution to the work, and approved it for publication.

## Conflict of Interest

The authors declare that the research was conducted in the absence of any commercial or financial relationships that could be construed as a potential conflict of interest.
