# Supplementary material for: Transitioning Out of the Coronavirus Lockdown: A Framework for Evaluating Zone-Based Social Distancing
Source: Front Public Health. 2020 Jun 8;8:266. doi: 10.3389/fpubh.2020.00266 (PMC7298272; doi:10.3389/fpubh.2020.00266)
Supplement: Supplementary file 1 [file Data_Sheet_1.pdf]

frontiers

Supplementary Material

1 Technical Appendices

1.1 Model and Results

In this section we present the formal analysis of our model. We first consider the SIR model on a random network with n nodes, with arbitrary degree distribution, which includes high degree nodes known as superspreaders, subject to the requirement that the variance of the nodal degrees is finite, and the nodes have average degree d (*1,2*). Let $h=1-1/d+Var(d)/d^2$. In the common model where the degree distribution is Poisson with mean d, we note that Var(d)=d, so h=1, the standard result.

We assume that we start with all but one node in the S (susceptible) state and a single node in the I (infected) state. In each period an infected node will infect its susceptible neighbor with probability b and become R (removed) with probability g, which implies that the average time in an infectious state is T=1/g. This implies that the transmissibility q=b/(b+g) which is the probability that an infected node infects its neighbor overall. For large n and d the following result is known:

Result (*1*): Let R_0= hqd, then if $R_0<1$, the number of infected nodes is less than $1/(1-R_0)$ while if $R_0>1$ the expected number of infected nodes is given by $nf(R_0)$ where $f(R_0)$ is an increasing function of R_0 which approaches 1 as R_0 approaches infinity.

This result is proven in (*1*), where our formulation is a direct computation of equation 23 in that paper.

In the common case where the degree distribution is Poisson with mean d, this reduces to the well-known relation R_0= hqd, but allows for generalizations to other distributions such as those with superspreaders.

Next consider an SIR model on a random zonal network. We assume there are m zones, which are groups of nodes, each of size s=n/m. Now we assume that each node has d_i edges to nodes in its own zone and d_o edges to nodes in other zones, where $d=d_i+d_o$. Define h_i and h_o as for h above and let $a_d=h_o/h$. Let $C_R= d_o/d$. We now consider the zone meta-network where each node is an entire zone of the original network. Thus, the zone meta-network has m nodes and we will apply the above result to it.

Assume a zonal isolation procedure, where we isolate a zone as soon as a node within that zone becomes symptomatic. Given an isolated zone with k infected people where the i'th person was infectious for time t_i before isolation, define the effective external infectivity time to be $I_T = (t_1+t_2+\ldots t_k)/T$. For large n and m, the dynamics on the zonal meta-network behave like an SIR network with degree $d_Z=sd_o$ and transmission probability $q_Z=I_Tq/s$.

Combining all of these we see that the inter-zonal reproduction number of the zone meta-network (analogous to the basic reproduction number in the simple SIR model),

$R_Z = d_Zq_Z=a_dR_0C_RI_T$.

Thus, the zonal reproduction satisfies the above proposition for the zone meta-network. In the main text we assume that d and d_o are just scaled versions of the same distribution so a_d=1. Formally, one can apply the results in (*1*) to show the correctness of this argument.

In the main text of the paper we consider the addition of a social distancing policy using the "truncation ratio" (T_R), which captures the ratio of social contacts under a zonal social distancing policy, relative to the unconstrained social structure (i.e., no distancing requirements or social pressures of any kind). In this case we see that

$R_Z = T_R a_d R_0 C_R I_T$,

since the number of contacts is reduced by T_R.

1.2 Simulation

In this simulation we assume that the nodes are on a 2-dimensional integer grid. In the distance-based model an infected node randomly chooses 980 local nodes that are within 100 city blocks with probability and 20 non-local nodes that are between 100 and 200 city blocks away. It then passes on the infection to each of these nodes with probability 0.5/1000.

In the zone-based protocol we divide the integer grid into fixed 200x200 node square zones. In this model an infected node randomly chooses 980 local nodes that are within its zone with probability 0.95 and 20 non-local nodes that are in adjacent zones.

In both models an infected node passes on the infection with probability 0.5/1000 to each of the selected nodes.

We assume that the time from infection to infectious is 2 days, and the time from infection to symptomatic is 7 days. We assume that a zone is isolated immediately once a node becomes symptomatic, so if the person becomes infected at the beginning of day 1 then isolation of zones for both models occurs at the beginning of day 8.

We initialize the simulation with 1 infected node and the remaining nodes are all susceptible. We iterate the network SIR model until we either infect a non-local node or the infection dies out.

Thus, in both cases each node has d=1000 neighbors and if we use a reasonable estimate of the empirical value T=10 we get a probability of transmission of q=(0.5/1000)*10, yielding we R_0=5. For the zone-based model we get R_Z=5. Computing R_Z is more difficult. By construction a_d=1 and C_R=0.02. Numerically we compute I_T=4.27, yielding R_Z=1*5*0.02*4.27=0.427.

We ran each simulation 10,000 times and found that the distance-based model fails to contain the virus about 27% of the time while the zone-based social model only fails about 6% of the time. If we enlarge the lockdown region by a factor of about 2.5 (in area) then the distance-based lockdown achieves the same performance as the zone-based lockdown according to this metric.

1.3 Sample Computation of I_T

Consider a policy where we test for people that show symptoms of COVID-19 and isolate a zone if any person in that zone tests positive for the virus.

For example, assume that a Person 1 who is infected at the start of Day 1 becomes infectious two days later (at the start of Day 3) and symptomatic at the start of Day 8, thereby triggering the isolation of the zone from all other zones at the start of day 8.

During this time span, assume Person 1 infects a new person in days 3,4 and 5, and– call these newly infected persons 2,3,4. Person 2 becomes infectious at the start of day 5 and infects person 5 that day. Then $t_1=5$, $t_2=3$, $t_3=2$, $t_4=1$, $t_5=1$. Thus, $I_T=12/14=0.86$ where we used T=14 as a reasonable value, based on WHO estimates for mild cases (*3*).

2. References

1. M. E. Newman, Spread of epidemic disease on networks. *Physical review E*. 66, 016128 (2002). https://journals.aps.org/pre/abstract/10.1103/PhysRevE.66.016128
2. "How a Premier U.S. Drug Company Became a Virus 'Super Spreader'." *NY Times*, 12 April 2020; https://www.nytimes.com/2020/04/12/us/coronavirus-biogen-boston-superspreader.html
3. World Health Organization, *Report of the WHO-China Joint Mission on Coronavirus Disease 2019 (COVID-19)* (WHO, 2020); https://www.who.int/docs/default-source/coronaviruse/who-china-joint-mission-on-covid-19-final-report.pdf
